# Supplementary material for: A Likelihood Approach for Real-Time Calibration of Stochastic Compartmental Epidemic Models
Source: PLoS Comput Biol. 2017 Jan 17;13(1):e1005257. doi: 10.1371/journal.pcbi.1005257 (PMC5240920; doi:10.1371/journal.pcbi.1005257)
Supplement: S1 File — (TAR.GZ) [file pcbi.1005257.s014.tar.gz › HSPH_Online-SI-Revision/Additions/IPoi10/n100-extreme_noisy/n100-extreme_noisy_BENCH2_table-4.pdf]

1	newly 3 weeks spec	10775.3	9329.77	{4053., 12304.}	23.4239	0.153161	newly 3 weeks cumul	25108.6	21191.6	{7036., 32797.}	32.2169	0.211963	newly 5 weeks spec	10934.9	10627.2	{8627., 12217.}	11.769	0.0572798	newly 5 weeks cumul	47514.6	42371.9	{24261., 53352.}	18.7022	0.110853	newly 58 weeks cumul	88201.8	84731.1	{78782., 89249.}	4.43677	0.0200751
2	newly 3 weeks spec	8383.	8201.92	{5750., 9778.}	15.2275	0.0723674	newly 3 weeks cumul	21608.	24987.7	{20228., 28599.}	21.5253	0.0889425	newly 5 weeks spec	8027.	6550.41	{3785., 9361.}	25.8224	0.140443	newly 5 weeks cumul	38163.	39035.1	{29148., 44909.}	13.6161	0.061081	newly 54 weeks cumul	72415.	65269.7	{43696., 84436.}	18.5465	0.0927988
3	newly 3 weeks spec	12915.	11371.3	{6596., 13267.}	13.2857	0.0924067	newly 3 weeks cumul	29820.	28048.3	{11709., 36948.}	24.1252	0.149704	newly 5 weeks spec	11421.	10498.2	{7546., 12986.}	15.3555	0.0813539	newly 5 weeks cumul	54424.	50093.8	{35011., 56657.}	10.6463	0.0678399	newly 59 weeks cumul	84212.	83448.5	{73209., 90574.}	5.63868	0.0251565
4	newly 3 weeks spec	15590.	4648.82	{888., 9047.}	70.1808	0.679534	newly 3 weeks cumul	27674.	7483.11	{1309., 14986.}	72.9598	0.738244	newly 5 weeks spec	15345.	12368.7	{5914., 16147.}	21.6059	0.149687	newly 5 weeks cumul	61225.	28400.6	{9668., 45456.}	53.6128	0.414634	newly 63 weeks cumul	88766.	93153.6	{92069., 93930.}	4.98974	0.0211256
5	newly 3 weeks spec	10521.	8665.34	{2915., 12434.}	29.6245	0.201182	newly 3 weeks cumul	21016.	18644.3	{4999., 31067.}	40.4915	0.253492	newly 5 weeks spec	12782.	11075.4	{8668., 12819.}	13.5546	0.0813387	newly 5 weeks cumul	46785.	40195.9	{19636., 53421.}	22.6563	0.14274	newly 60 weeks cumul	85329.	84634.7	{79741., 88146.}	3.18015	0.0141157
6	newly 3 weeks spec	10702.	10220.7	{6150., 11827.}	13.5956	0.0767383	newly 3 weeks cumul	23963.	27215.9	{11811., 33999.}	32.6136	0.151431	newly 5 weeks spec	11271.	9133.78	{6322., 11685.}	20.3374	0.108988	newly 5 weeks cumul	47247.	46407.9	{32639., 52257.}	11.7713	0.0606599	newly 58 weeks cumul	86171.	77729.8	{65479., 88561.}	10.6546	0.0516384
7	newly 3 weeks spec	11555.	6404.85	{1191., 9783.}	44.5707	0.355639	newly 3 weeks cumul	26529.	14438.3	{2170., 23731.}	46.1359	0.391832	newly 5 weeks spec	11237.	8533.2	{3864., 10236.}	24.0616	0.153399	newly 5 weeks cumul	49918.	30734.8	{8316., 43660.}	38.4295	0.281789	newly 56 weeks cumul	84780.	86502.8	{81547., 90531.}	4.13081	0.0177439
8	newly 3 weeks spec	11018.	8839.9	{3093., 11821.}	24.4397	0.185552	newly 3 weeks cumul	24447.	21376.1	{5369., 33562.}	36.7313	0.252164	newly 5 weeks spec	11207.	9670.98	{7344., 11726.}	15.7388	0.0900406	newly 5 weeks cumul	47781.	40735.5	{19334., 51902.}	20.9247	0.139707	newly 58 weeks cumul	87124.	83168.7	{73672., 89589.}	6.08166	0.027937
9	newly 3 weeks spec	10870.	5001.83	{1188., 9509.}	53.992	0.46849	newly 3 weeks cumul	22125.	10247.3	{2048., 21686.}	55.3203	0.499023	newly 5 weeks spec	12694.	8455.43	{4029., 10886.}	33.3903	0.229761	newly 5 weeks cumul	47679.	25573.1	{8382., 43219.}	46.3994	0.362643	newly 58 weeks cumul	88989.	91659.5	{87972., 93489.}	3.5201	0.0150171
10	newly 3 weeks spec	15442.	8155.11	{1029., 13978.}	47.1888	0.397362	newly 3 weeks cumul	29673.	14651.3	{1557., 27486.}	51.5059	0.453029	newly 5 weeks spec	11765.	13323.	{5691., 15622.}	29.8251	0.139323	newly 5 weeks cumul	57244.	39719.7	{9769., 56566.}	31.0412	0.22352	newly 62 weeks cumul	72720.	86894.	{83060., 90384.}	19.4912	0.0770677
11	newly 3 weeks spec	9197.	9451.71	{6715., 11182.}	16.7898	0.0850013	newly 3 weeks cumul	23145.	26412.7	{13953., 32017.}	31.3494	0.147079	newly 5 weeks spec	8893.	8093.76	{5440., 10938.}	21.6513	0.106965	newly 5 weeks cumul	41559.	43552.9	{34316., 49402.}	15.2192	0.0721449	newly 56 weeks cumul	75767.	71550.	{56679., 84629.}	12.058	0.0567653
12	newly 3 weeks spec	11654.	4846.5	{1554., 8284.}	58.4134	0.469023	newly 3 weeks cumul	24296.	10058.4	{2886., 18763.}	58.7013	0.489245	newly 5 weeks spec	12745.	8074.21	{4516., 10153.}	36.648	0.230494	newly 5 weeks cumul	50388.	24712.3	{10184., 38593.}	50.956	0.370825	newly 57 weeks cumul	88506.	88528.	{85552., 90446.}	1.71983	0.00751441
13	newly 3 weeks spec	8371.	8425.7	{4907., 9805.}	17.0236	0.0910398	newly 3 weeks cumul	19985.	22743.3	{9571., 28460.}	33.2993	0.156351	newly 5 weeks spec	8948.	8146.22	{6079., 9829.}	14.344	0.074272	newly 5 weeks cumul	38014.	39414.	{25274., 44946.}	16.3892	0.0817576	newly 55 weeks cumul	83218.	78679.8	{64875., 90805.}	10.1234	0.0477103
14	newly 3 weeks spec	11434.	1816.15	{318., 4146.}	84.1162	1.01611	newly 3 weeks cumul	18644.	3298.46	{584., 7723.}	82.3082	0.971247	newly 5 weeks spec	15310.	4849.21	{1437., 8742.}	68.3265	0.638346	newly 5 weeks cumul	50732.	11259.5	{2776., 23124.}	77.806	0.823101	newly 60 weeks cumul	70240.	93332.7	{92524., 93832.}	32.8768	0.123443
15	newly 3 weeks spec	13412.	8025.7	{3880., 12479.}	40.1603	0.297369	newly 3 weeks cumul	27516.	15930.1	{6613., 28113.}	43.6468	0.337583	newly 5 weeks spec	12056.	11885.1	{10831., 13203.}	9.78446	0.0652908	newly 5 weeks cumul	54113.	38485.4	{24379., 53358.}	29.0064	0.195067	newly 60 weeks cumul	75951.	89251.6	{86058., 91245.}	17.5121	0.0699687
16	newly 3 weeks spec	15726.	8233.85	{3310., 11570.}	47.6418	0.345955	newly 3 weeks cumul	30526.	17035.7	{5673., 25848.}	44.991	0.340377	newly 5 weeks spec	14011.	11246.5	{8897., 12527.}	19.7307	0.111609	newly 5 weeks cumul	61514.	38639.1	{21021., 50264.}	37.1865	0.240737	newly 60 weeks cumul	88752.	88159.9	{85654., 90608.}	2.03665	0.00901399
17	newly 3 weeks spec	9053.	8148.51	{3593., 10213.}	20.4237	0.134592	newly 3 weeks cumul	21971.	20165.5	{6744., 29102.}	29.2636	0.185675	newly 5 weeks spec	9419.	8834.11	{6696., 10145.}	12.2482	0.0645421	newly 5 weeks cumul	40887.	37826.3	{20461., 46244.}	18.106	0.10905	newly 55 weeks cumul	79924.	82363.3	{73170., 89657.}	7.27381	0.0311814
18	newly 3 weeks spec	8959.	6457.35	{2466., 9922.}	32.885	0.2246	newly 3 weeks cumul	19558.	14493.7	{4614., 25658.}	39.7997	0.268307	newly 5 weeks spec	10392.	8831.26	{6317., 10233.}	15.0247	0.0845078	newly 5 weeks cumul	40376.	31222.1	{14977., 45133.}	27.5335	0.17231	newly 56 weeks cumul	75084.	86409.6	{80637., 90163.}	15.3004	0.0615125
19	newly 3 weeks spec	11412.	6762.	{2025., 10245.}	40.7466	0.296293	newly 3 weeks cumul	23492.	14270.4	{3462., 23231.}	42.4462	0.318642	newly 5 weeks spec	12862.	9814.68	{5963., 11245.}	23.6924	0.13631	newly 5 weeks cumul	49674.	32732.7	{13008., 45072.}	34.105	0.225702	newly 58 weeks cumul	89565.	83970.8	{80597., 86569.}	6.24595	0.0282148
20	newly 3 weeks spec	15824.	877.623	{74., 2017.}	94.4538	1.50416	newly 3 weeks cumul	21990.	1471.85	{119., 3343.}	93.3067	1.42241	newly 5 weeks spec	20039.	3435.87	{411., 6937.}	82.8541	0.971702	newly 5 weeks cumul	66304.	6731.89	{707., 14213.}	89.8469	1.21386	newly 63 weeks cumul	91232.	91765.8	{91084., 92200.}	0.822889	0.0035637
21	newly 3 weeks spec	11565.	7477.95	{1897., 11749.}	37.4613	0.304192	newly 3 weeks cumul	23774.	14973.2	{3058., 25888.}	44.0614	0.360143	newly 5 weeks spec	12361.	11252.5	{7015., 13140.}	14.1414	0.096508	newly 5 weeks cumul	49979.	36151.6	{13864., 51346.}	29.6481	0.215418	newly 60 weeks cumul	80337.	89495.1	{87109., 91673.}	11.4354	0.0468571
22	newly 3 weeks spec	13043.	6676.93	{1312., 12013.}	48.9853	0.409019	newly 3 weeks cumul	25673.	12383.9	{2056., 24023.}	53.2617	0.463152	newly 5 weeks spec	13126.	11743.1	{5935., 13935.}	14.7428	0.0995896	newly 5 weeks cumul	53143.	33812.3	{10991., 51280.}	36.797	0.265661	newly 61 weeks cumul	81637.	91061.	{89231., 92239.}	11.5437	0.0474049
23	newly 3 weeks spec	8971.	8583.67	{5949., 9735.}	12.1055	0.0796428	newly 3 weeks cumul	22691.	23068.9	{12165., 28283.}	22.0104	0.128018	newly 5 weeks spec	8717.	8189.58	{6697., 9588.}	12.0254	0.0638811	newly 5 weeks cumul	40575.	39912.	{29430., 44446.}	11.9511	0.0715706	newly 54 weeks cumul	74886.	79208.7	{68678., 89483.}	9.78086	0.0407721
24	newly 3 weeks spec	9753.	6220.27	{1364., 8976.}	36.222	0.295818	newly 3 weeks cumul	22644.	15662.9	{2594., 25801.}	37.4116	0.31899	newly 5 weeks spec	10377.	7180.18	{3459., 9064.}	30.8068	0.196754	newly 5 weeks cumul	43672.	29717.	{8291., 41248.}	31.9542	0.244964	newly 54 weeks cumul	86096.	80814.3	{70782., 88531.}	7.44476	0.0348504
25	newly 3 weeks spec	14475.	5211.54	{765., 8876.}	63.9963	0.578993	newly 3 weeks cumul	28620.	11134.9	{1441., 20065.}	61.0941	0.567577	newly 5 weeks spec	13481.	7968.24	{2546., 10341.}	40.8928	0.287352	newly 5 weeks cumul	57956.	25853.7	{5409., 40494.}	55.3908	0.449587	newly 57 weeks cumul	86463.	88000.1	{84829., 90697.}	3.07031	0.0132243
26	newly 3 weeks spec	8715.	8848.92	{6305., 10276.}	15.9537	0.0819283	newly 3 weeks cumul	21621.	23826.1	{12760., 29644.}	29.6928	0.141008	newly 5 weeks spec	8698.	8321.87	{6127., 10071.}	14.8967	0.069995	newly 5 weeks cumul	39423.	41047.	{31047., 46225.}	15.4124	0.0734235	newly 55 weeks cumul	81661.	77097.1	{64207., 88166.}	9.74552	0.0454583
27	newly 3 weeks spec	9364.	9061.39	{6592., 11124.}	14.3618	0.067993	newly 3 weeks cumul	24919.	28563.5	{22026., 32961.}	21.8371	0.0922332	newly 5 weeks spec	8776.	6707.66	{3860., 10871.}	32.5916	0.178039	newly 5 weeks cumul	43318.	43270.9	{33829., 49997.}	11.4533	0.0520424	newly 56 weeks cumul	79621.	65420.3	{44684., 88252.}	21.8708	0.1127
28	newly 3 weeks spec	11755.	11292.5	{6928., 13979.}	21.4921	0.130898	newly 3 weeks cumul	27332.	26036.1	{11807., 38275.}	32.5217	0.194289	newly 5 weeks spec	11093.	11458.5	{8080., 13994.}	17.9076	0.0800711	newly 5 weeks cumul	50746.	49658.4	{36647., 58900.}	16.7827	0.092028	newly 60 weeks cumul	87654.	86387.8	{79057., 91494.}	4.43598	0.0198165
29	newly 3 weeks spec	8104.	7051.43	{1670., 9659.}	27.6561	0.202243	newly 3 weeks cumul	20099.	17472.3	{3128., 27536.}	35.7088	0.25712	newly 5 weeks spec	8778.	8037.82	{4610., 9630.}	15.2008	0.0950596	newly 5 weeks cumul	37699.	33319.7	{10696., 43905.}	24.603	0.168417	newly 54 weeks cumul	75236.	84401.5	{75191., 91704.}	13.4994	0.0545938
30	newly 3 weeks spec	13462.	11307.9	{3720., 16345.}	29.075	0.182179	newly 3 weeks cumul	25138.	21287.1	{5631., 34753.}	38.01	0.235751	newly 5 weeks spec	14527.	14740.	{12621., 16729.}	10.3232	0.0455719	newly 5 weeks cumul	55394.	50485.1	{28465., 63541.}	18.3026	0.0967974	newly 63 weeks cumul	91285.	90079.7	{88366., 92035.}	1.63526	0.00720705
31	newly 3 weeks spec	13467.	2556.06	{275., 4816.}	81.0198	0.973259	newly 3 weeks cumul	21982.	3908.1	{407., 7107.}	82.2214	1.01647	newly 5 weeks spec	17282.	9995.19	{2433., 15473.}	42.1642	0.390089	newly 5 weeks cumul	58272.	19568.3	{3546., 32771.}	66.4191	0.662579	newly 64 weeks cumul	87865.	89929.8	{91058., 91567.}	5.52458	0.111103
32	newly 3 weeks spec	11691.	7770.73	{1325., 13639.}	40.1295	0.331566	newly 3 weeks cumul	23586.	14806.1	{2125., 28872.}	47.2991	0.398446	newly 5 weeks spec	12032.	12132.3	{6571., 14526.}	20.5683	0.109735	newly 5 weeks cumul	48919.	37525.7	{11813., 56312.}	29.915	0.209764	newly 61 weeks cumul	74173.	91633.8	{89036., 93565.}	23.5407	0.0916818
33	newly 3 weeks spec	9366.	10306.6	{6516., 12511.}	27.7572	0.132544	newly 3 weeks cumul	20595.	24459.7	{12117., 35285.}	42.6248	0.190315	newly 5 weeks spec	10722.	10641.2	{8011., 12491.}	13.3998	0.0624159	newly 5 weeks cumul	42102.	46182.3	{33927., 54028.}	22.3122	0.101772	newly 59 weeks cumul	87634.	84115.5	{76052., 89604.}	5.20518	0.0237822
34	newly 3 weeks spec	6522.	7985.54	{4598., 9605.}	36.0968	0.152909	newly 3 weeks cumul	16514.	20929.1	{9220., 27786.}	46.0998	0.193324	newly 5 weeks spec	7135.	8186.42	{5798., 9634.}	22.2073	0.0894335	newly 5 weeks cumul	30872.	37439.9	{24186., 44071.}	32.5398	0.134984	newly 54 weeks cumul	67088.	79991.9	{65688., 90008.}	20.9246	0.0812113
35	newly 3 weeks spec	10120.	7682.92	{2668., 11785.}	31.6074	0.232557	newly 3 weeks cumul	20852.	16664.	{4603., 30145.}	39.4507	0.277746	newly 5 weeks spec	12158.	10205.7	{7924., 11869.}	16.0577	0.096455	newly 5 weeks cumul	45344.	36289.4	{17398., 51472.}	25.5747	0.171068	newly 59 weeks cumul	90553.	86460.1	{80527., 89610.}	4.51994	0.0204292
36	newly 3 weeks spec	8518.	8561.28	{5350., 11529.}	24.0948	0.115106	newly 3 weeks cumul	20967.	28158.2	{20736., 33692.}	41.6763	0.164537	newly 5 weeks spec	8858.	5804.84	{3206., 9274.}	38.7389	0.236736	newly 5 weeks cumul	39044.	41150.6	{28750., 51210.}	20.9151	0.0934694	newly 57 weeks cumul	75101.	58781.5	{39052., 80297.}	25.4994	0.138682
37	newly 3 weeks spec	14436.	7962.21	{2354., 13957.}	45.5363	0.375515	newly 3 weeks cumul	27190.	14531.8	{3665., 28045.}	50.0702	0.426174	newly 5 weeks spec	14344.	13163.4	{10291., 15311.}	12.9402	0.0862281	newly 5 weeks cumul	57920.	39080.1	{19385., 56650.}	33.0144	0.232753	newly 62 weeks cumul	85971.	86238.2	{82686., 92405.}	3.55085	0.0152573
38	newly 3 weeks spec	14671.	1876.29	{260., 4592.}	87.2109	1.14423	newly 3 weeks cumul	23153.	3198.24	{412., 8160.}	86.1865	1.12216	newly 5 weeks spec	16782.	5714.88	{1147., 11126.}	65.9464	0.636578	newly 5 weeks cumul	59731.	12413.1	{2126., 27126.}	79.2184	0.883412	newly 62 weeks cumul	80717.	89008.8	{87551., 93389.}	10.2726	0.0423799
39	newly 3 weeks spec	8107.	6423.	{1297., 10178.}	33.5279	0.250333	newly 3 weeks cumul	18981.	14368.2	{2500., 25745.}	39.502	0.301688	newly 5 weeks spec	8590.	8701.95	{4069., 10416.}	20.7428	0.120213	newly 5 weeks cumul	36334.	30897.4	{8987., 45713.}	29.351	0.199475	newly 56 weeks cumul	64192.	87626.5	{82157., 91351.}	36.5069	0.134809
40	newly 3 weeks spec	9375.	10866.6	{8440., 12344.}	20.1858	0.080497	newly 3 weeks cumul	23924.	31332.7	{24745., 35212.}	33.3962	0.124386	newly 5 weeks spec	8935.	8118.2	{5061., 11503.}	24.6767	0.122266	newly 5 weeks cumul	42535.	49226.5	{42669., 53171.}	17.7315	0.070785	newly 57 weeks cumul	74746.	71967.2	{55271., 84969.}	12.7063	0.0590829
41	newly 3 weeks spec	9002.	9223.52	{2614., 12505.}	35.3108	0.198514	newly 3 weeks cumul	21615.	21403.6	{4518., 34360.}	42.4126	0.251547	newly 5 weeks spec	8585.	10361.4	{7715., 12431.}	28.8825	0.114139	newly 5 weeks cumul	39635.	42056.7	{17703., 54042.}	30.6476	0.153064	newly 58 weeks cumul	64657.	86032.6	{78250., 91363.}	33.06	0.123241
42	newly 3 weeks spec	13145.	11071.5	{2291., 15706.}	28.5378	0.21066	newly 3 weeks cumul	25907.	22686.5	{3456., 37358.}	39.4882	0.273849	newly 5 weeks spec	11817.	12910.5	{9594., 15594.}	20.9465	0.0932174	newly 5 weeks cumul	52038.	48671.7	{19371., 62604.}	21.9532	0.133006	newly 62 weeks cumul	70332.	87838.5	{84339., 90422.}	24.8912	0.0963297
43	newly 3 weeks spec	10255.	6438.58	{2337., 9512.}	37.2152	0.282289	newly 3 weeks cumul	22579.	14928.8	{4350., 24188.}	36.9865	0.294988	newly 5 weeks spec	11209.	8267.69	{5849., 9724.}	26.2406	0.159251	newly 5 weeks cumul	45260.	30781.6	{13989., 42789.}	31.9894	0.225565	newly 55 weeks cumul	87509.	86981.2	{81743., 91551.}	3.68419	0.0161968
44	newly 3 weeks spec	15502.	11920.2	{6130., 14510.}	23.1054	0.150676	newly 3 weeks cumul	33206.	27000.6	{10445., 39460.}	27.7799	0.186777	newly 5 weeks spec	12451.	11947.	{9071., 14279.}	14.0291	0.067336	newly 5 weeks cumul	61309.	51797.3	{35286., 60544.}	15.5143	0.0916411	newly 61 weeks cumul	87960.	88425.5	{82448., 93077.}	4.0216	0.0175871
45	newly 3 weeks spec	16177.	5543.28	{581., 11181.}	65.7336	0.657529	newly 3 weeks cumul	31598.	10851.5	{972., 24191.}	65.6577	0.684828	newly 5 weeks spec	13010.	9458.19	{2652., 12445.}	27.3006	0.218972	newly 5 weeks cumul	61499.	28071.8	{4962., 48811.}	54.354	0.471118	newly 60 weeks cumul	80329.	89620.7	{86313., 91093.}	11.567	0.0474512
46	newly 3 weeks spec	10769.	4603.24	{558., 8436.}	57.4746	0.544274	newly 3 weeks cumul	20202.	8662.15	{876., 16575.}	58.9971	0.56995	newly 5 weeks spec	14108.	9060.06	{2542., 12332.}	35.7807	0.284805	newly 5 weeks cumul	48516.	24669.9	{4648., 40184.}	49.1624	0.423154	newly 60 weeks cumul	82843.	88870.5	{87506., 89966.}	7.27587	0.0304626
47	newly 3 weeks spec	12425.	8205.62	{1473., 13115.}	38.4878	0.316113	newly 3 weeks cumul	24055.	14762.7	{2199., 24665.}	44.5113	0.376518	newly 5 weeks spec	13477.	13472.	{8240., 15700.}	16.5093	0.0935013	newly 5 weeks cumul	52391.	39996.7	{14211., 54893.}	27.0134	0.190855	newly 62 weeks cumul	79732.	89494.7	{88293., 90626.}	12.2444	0.0501285
48	newly 3 weeks spec	12536.	3364.42	{282., 6578.}	73.162	0.802674	newly 3 weeks cumul	21158.	5758.31	{430., 11508.}	73.2351	0.814585	newly 5 weeks spec	16548.	8791.48	{1668., 13243.}	46.8729	0.404381	newly 5 weeks cumul	54873.	20477.2	{2821., 35358.}	62.6825	0.598578	newly 62 weeks cumul	90499.	92095.3	{91503., 92653.}	1.79361	0.00771528
49	newly 3 weeks spec	21183.	3615.62	{644., 7757.}	82.9315	0.947171	newly 3 weeks cumul	34718.	5580.89	{895., 12184.}	83.9251	0.985513	newly 5 weeks spec	14957.	11899.6	{5182., 16727.}	25.3459	0.188896	newly 5 weeks cumul	71735.	24879.9	{8003., 42882.}	65.3169	0.562078	newly 64 weeks cumul	87984.	91796.7	{90848., 93386.}	4.33339	0.0183982
50	newly 3 weeks spec	10269.	9148.53	{6495., 10685.}	13.2541	0.0842836	newly 3 weeks cumul	26490.	23304.9	{12590., 30645.}	21.735	0.135155	newly 5 weeks spec	9271.	9149.04	{7278., 10651.}	12.6579	0.0585545	newly 5 weeks cumul	46170.	41996.2	{31871., 48133.}	11.6844	0.0681425	newly 55 weeks cumul	81778.	83439.8	{73102., 91089.}	7.32174	0.0317676
